# Synthesis and structural evolution of Ba_1-3x_La_2x_Ti_1-3x_Bi_4x_O_3_ solid solutions (0.0 ≤ x ≤ 0.05)

**DOI:** 10.1016/j.heliyon.2024.e38463

**Published:** 2024-09-26

**Authors:** M.I. Valenzuela-Carrillo, M. Pérez-Labra, F.R. Barrientos-Hernández, J.A. Romero-Serrano, M. Reyes-Pérez, A. Cruz-Ramírez, M.U. Flores-Guerrero, J.C. Juárez-Tapia

**Affiliations:** a Academic Area of Earth Sciences and Materials. Autonomous University of Hidalgo State. Road Pachuca- Tulancingo Km 4.5 Mineral de la Reforma Zip Code 42184, Hidalgo Mexico; b Instituto Politécnico Nacional, ESIQIE, Metallurgy and Materials Department, Zacatenco, Zip Code 07738, Ciudad de México, México; c Industrial Electromechanics Area, Technological University of Tulancingo, 43642 Hidalgo, Mexico

**Keywords:** BaTiO_3_, Doping, Lanthanum, Bismuth, Characterization

## Abstract

In this study, the synthesis and structural evolution of pure BaTiO_3_ and BaTiO_3_ co-doped with La^3+^ and Bi^3+^, produced using the ball milling method and heat treatment, were analyzed. The starting materials included chemically pure precursor powders of BaCO_3_, TiO_2_, La_2_O_3,_ and Bi_2_O_3_. Stoichiometric calculations were performed using the Ba_1-3x_La_2x_Ti_1-3x_Bi_4x_O_3_ mechanism with concentrations x = 0.0, 0.0025, 0.0045, 0.006, 0.01, and 0.05 (mol %). The structural and morphological evolution of the samples was characterized by x-ray diffraction (XRD), Rietveld refinement, and high-resolution scanning electron microscopy (HRSEM-EDS). XRD and Rietveld refinement results indicated a tetragonal ferroelectric phase for samples with x ≤ 0.006. As doping levels increased, a peak disappearance was observed, corresponding to a phase transformation from tetragonal to cubic at x = 0.01. Additionally, a cubic secondary phase, BaBiO₃, was detected at x = 0.05, indicating that the maximum solubility of La³⁺ and Bi³⁺ ions in BaTiO_3_ was exceeded at this concentration. The concentration x = 0.0025 was found to exhibit the maximum tetragonality among the samples studied, with a value of 1.0087, greater than the tetragonality for pure BaTiO_3_, 1.0083. The HRSEM-EDS analysis revealed that for samples with x ≤ 0.006, the microstructure consisted of faceted shape grains with mean grain size in the range of 362.5 nm–488.3 nm. Punctual microanalysis and elemental distribution mapping of the doped samples showed that the grains were composed of Ba, Ti, O, Bi, and La, which were homogeneously distributed, confirming the incorporation of the dopant elements into the BaTiO_3_ structure.

## 1 Introduction

Perovskite-phase metal oxides exhibit various interesting physical properties, such as ferroelectricity, piezoelectricity, and pyroelectricity. Among these metal oxides, BaTiO_3_ is particularly significant and widely used in capacitors, multilayer capacitors (MLCs), and energy storage devices due to its attractive dielectric and ferroelectric characteristics [[1], [2], [3], [4]]. At room temperature, BaTiO_3_ exists in the tetragonal phase, which transforms to the cubic phase at 130 °C. The phase transformation from tetragonal to cubic phase has received wide attention for its correlation with the shift from ferroelectric to paraelectric behavior of BaTiO_3_ [5,6]. It is known that the electrical properties of BaTiO_3_ ceramics are strongly influenced by its crystal structure. It can be easily shown that the high dielectric constant of BaTiO_3_ powder is correlated with its high tetragonality which originates from the tetragonal distortion of the lattice [7,8].

In this work, the effect of incorporating different amounts of La^3+^ and Bi^3+^ in the structure of BaTiO_3_ was investigated. Our focus is on the phase evolution, morphology, and development of secondary phases. To achieve this, we synthesize and characterize Ba_1-3x_La_2x_Ti_1-3x_Bi_4x_O_3_ ceramics, where x represents the concentration of dopant ions (mol %). Substituting ions in BaTiO_3_ is very important, particularly in the electronic device industry. Ionic size is a significant factor in the incorporation of ions in BaTiO_3_ ceramics. Previous studies have shown that trivalent ions, such as La^3+^ and Bi^3+^ can act as donors or acceptors in the BaTiO_3_ structure, and the final properties of BaTiO_3_ depend on the size of the ion used [9,10]. Specifically, La^3+^ and Bi^3+^ are cations highly soluble in BaTiO_3_, and it is for this reason that they are used individually to improve the physical properties of this compound [[11], [12], [13], [14], [15]]. It is considered that the valence state and the radius of La^3+^(1.06 Å) and Bi^3+^ (1.20 Å) ions are intermediate between those of Ba^2+^ ion (1.42 Å) and Ti^4+^ ion (0.61 Å). It is then expected that La^3+^ and Bi^3+^ can occupy either barium or titanium sites, depending on the Ba/Ti mole ratio [16,17]. The incorporation of the dopant ions into the BaTiO_3_ lattice induces modifications in the crystalline structure of the ceramic. In this study, the tetragonality of each sample was systematically measured to assess its evolution as a function of dopant concentration, with the aim of determining the specific concentration at which maximum tetragonality is achieved. The tetragonality (*c/a*) is measured from the lattice parameters of the *c*- and *a*-axes based on the XRD results. The MLCs industry prefers BaTiO_3_ powders with a tetragonality greater than 1.008 [18]; therefore, higher or equal tetragonality values are expected to be found.

To achieve certain characteristics in a material, the synthesis method used can be varied. There are different methods available for this purpose, including conventional solid-state reaction, non-conventional methods such as the Pechini process, the sol-gel procedure, or special mechanical treatment of initial powders [19]. In this investigation, the ball milling method was used employing zirconia balls, which is part of the solid-state synthesis methods and has been proven successful in synthesizing nano-crystalline oxide powders, such as perovskite ferroelectrics [20]. The ball charge was distributed by three sizes of zirconia balls considering the Gaudin-Schuman distribution [21].

## 2 Materials and methods

La^3+^ and Bi^3+^ doped BaTiO_3_ ceramics were prepared according to the mechanism Ba_1-3x_La_2x_Ti_1-3x_Bi_4x_O_3_, with x = 0.0, 0.0025, 0.0045, 0.006, 0.01, and 0.05 (mol %), using the ball milling method and heat treatment. The starting precursors included Barium Carbonate (BaCO_3_, 99.0 % CAS: 513-77-9), Titanium Oxide (TiO_2_, 99.0 % CAS: 13463-67-7), Lanthanum Oxide (La_2_O_3_, 99.9 % CAS: 1312-81-8), and Bismuth Oxide (Bi_2_O_3_, 99.999 % CAS: 1312-81-8), which were dried in a muffle-type oven at 200 °C for 24 h prior to weighing. These precursor reagents were placed inside a cylindrical polypropylene container along with zirconia oxide balls of three different diameters (2.95 mm, 5.05 mm, and 6.48 mm), and acetone was used as a control medium. The mixture was ground for 6 h in a ball milling process and subsequently dried. The ball charge distribution for ball milling was calculated using the Gaudin-Schumann equation [21]:

(1) $$Y=100\times {\left(\frac{d}{d_{\max}}\right)}^{3.8}$$

where, *Y* is the cumulative contribution of a ball of smaller diameter, d is the ball diameter and d_max_ is the diameter of the largest ball.

The mixture was decarbonated at 900 °C for 12 h using an Al_2_O_3_ crucible as a container. The powder mixture obtained was sintered in in air atmosphere at 1200 °C for 6 h in a Thermolyne muffle using a heating ramp of 4 °C/min, and subsequently, it was ground for 30 min in an agate mortar. The crystalline evolution was evaluated and monitored using X-ray diffraction (diffractometer: Inel, model: Equinox 2000 Co Kα1, 110° curved detector, V 30 kV, 20 mA, λ = 1.789 Å, resolution: 0.095 FWHM). The particle size distribution and powder morphology were characterized through image processing of high-resolution scanning electron microscopy (HRSEM, JEOL 6701F, JEOL Ltd., Tokyo, Japan) images. The mean grain diameter was measured using the Feret measurement method and the ImageJ software [22]. The software 'FullProf' was used to set up and run Rietveld refinements for determining the structural parameters of the phases present in the samples. The pseudo-Voigt (pV) profile shape function was employed for peak shape modeling. The background was adjusted using a polynomial function of degree four. The reliability index parameters R_wp_ (weighted residual error) and R_exp_ (expected error) were utilized to monitor the minimization process and obtain the refinement fit value (χ^2^) [23,24]. The Gaussian pseudo-Voigt shape function was dependent on the diffraction angle, as indicated by the following equation:

(2) $$W_{G}^{2}={Utan}^{2}\theta +Vtan\theta +W+P/\cos^{2}\;\theta$$

where *U*, *V*, and *W* represent the parameters of the Caglioti equation and *P* denotes the coefficient to simulate an extended Gaussian Scherrer function [24,25].

## 3 Results and discussion

### 3.1 X-ray diffraction

**Fig. 1:**
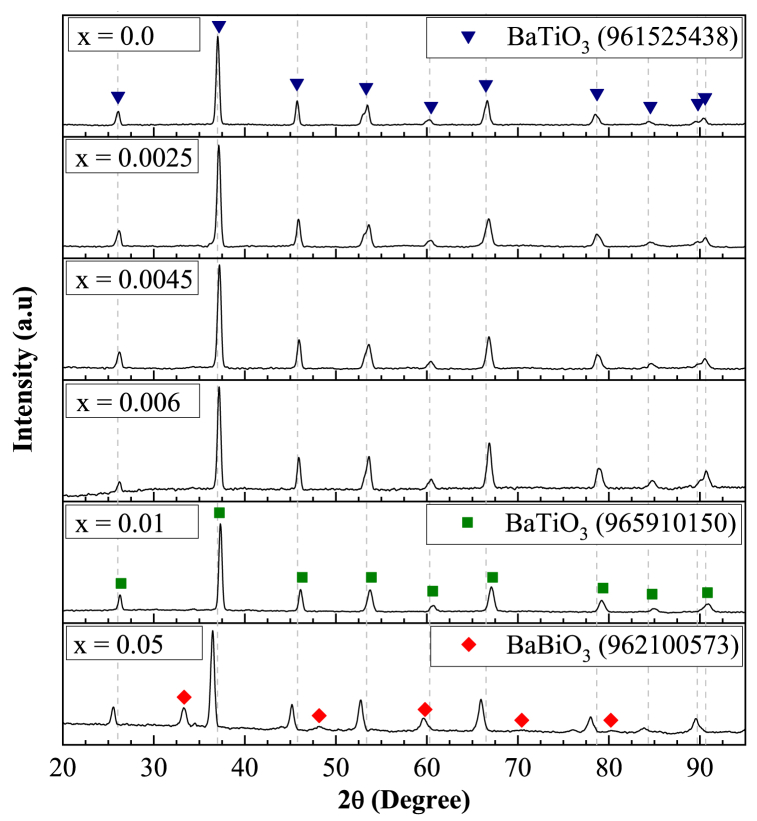
XRD patterns of the powder samples sintered at 1200 °C during 6 h in air atmosphere with different concentrations of x.

The XRD patterns obtained for the BaTiO_3_-based sintered powders are shown in Fig. 1, corresponding to the samples with compositions x = 0.0, 0.0025, 0.0045, 0.006, 0.01, and 0.05. For compositions where 0.0 ≤ x ≤ 0.006 of La^3+^ and Bi^3+^ (mol%), all the peaks observed in the diffractograms are similar to those present in the diffraction pattern for pure BaTiO_3_ in the tetragonal phase at room temperature (JCPDS 961525438, at positions 2θ ≈ 26.12, 37.06, 45.80, 53.49, 60.30, 66.67, 78.56, 84.42, 89.56, 90.44). However, for compositions where the amount of La^3+^ and Bi^3+^ exceeds x ≥ 0.01 (mol%), the BaTiO_3_ paraelectric cubic phase was identified (JCPDS 965910150, at positions 2θ ≈ 26.29, 37.33, 46.13, 53.74, 60.73, 67.10, 79.20, 84.97, 90.93).

**Fig. 2:**
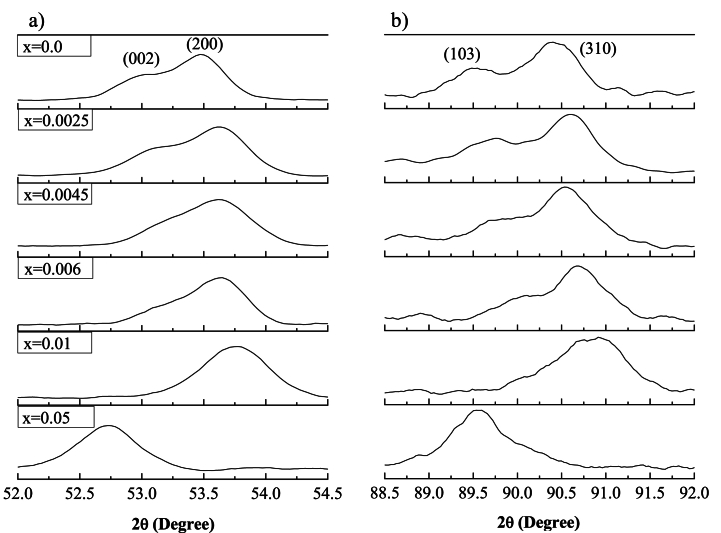
(a) zoom at 2θ ~ 52°–54.5°, and (b) 2θ ~ 88.5°–92° of the XRD patterns of the powder samples sintered at 1200 °C during 6 h in air atmosphere with different concentrations of x.

Fig. 2 shows the XRD peaks of the samples in the range from 2θ = 52°–54.5° and 2θ = 88.5°–92°. The crystallinity of BaTiO_3_ is characterized by the peak height and the splitting of the cubic (200) XRD reflection into two tetragonal (200)/(002) reflections. The splitting of (200)/(002) diffraction peaks was observed in Fig. 2 (a) in the samples where 0.0 ≤ x ≤ 0.006, indicating the existence of a tetragonal ferroelectric phase of BaTiO_3_ crystal. However, for the samples with x = 0.01 and x = 0.05, only one symmetric (200) peak was observed, indicating a symmetry transformation from the ferroelectric tetragonal phase to the paraelectric cubic phase as the amount of dopant was increased. This result is confirmed by Fig. 2 (b), where the transformation from the tetragonal to the cubic phase is again observed as the dopant concentration was increased, transitioning from the tetragonal reflections (103)/(310) to the cubic reflection (103).These results seem to agree with other authors who have reported that the tetragonal-cubic change associated to the ferroelectric–paraelectric phase transition takes place at room temperature for BaTiO_3_ doped with high concentrations of La^3+^ or Bi^3+^ [26,27].

No secondary phases were observed when the La^3+^ and Bi^3+^ content was increased from x = 0.0 to x = 0.01. However, a cubic secondary phase, identified as BaBiO_3_ (JCPDS 962100573), was found for the sample with x = 0.05 for La^3+^ and Bi^3+^ (mol%). This indicated that the maximum solubility of La^3+^ and Bi^3+^ ions into the BaTiO_3_ structure was exceeded for the sample with 0.05 mol%. It has been reported that BaBiO_3_ exhibits a distorted perovskite crystal structure with an ordered alternating arrangement of Bi^3+^ and Bi^5+^ ions [28,29]. Various studies have explored doping the compound with acceptors such as potassium, lead, and other additives, aiming for potential applications in high-temperature superconductors [[30], [31], [32], [33]]. The transition from semiconducting to superconducting behavior occurs upon doping, where the critical temperature typically varies depending on the type and concentration of dopants used. While doped BaBiO_3_ has primarily been investigated for superconducting applications, some researchers have also studied BaBiO_3_-doped BaTiO_3_ solid solutions and these studies explore their potential use in positive temperature coefficient of resistivity (PTCR) devices [34,35]. The emergence of a secondary phase may be a contributing factor to the observation of a cubic phase at concentrations exceeding x = 0.0100. This phase competes with the tetragonal phase for stability, potentially leading to a transformation from the tetragonal to the cubic structure at lower temperatures. This occurs because the presence of the secondary phase alters the total energy of the system, making the cubic phase more favorable under certain conditions, as reported in studies of BaTiO_3_ doped with various elements [36,37].

**Fig. 3:**
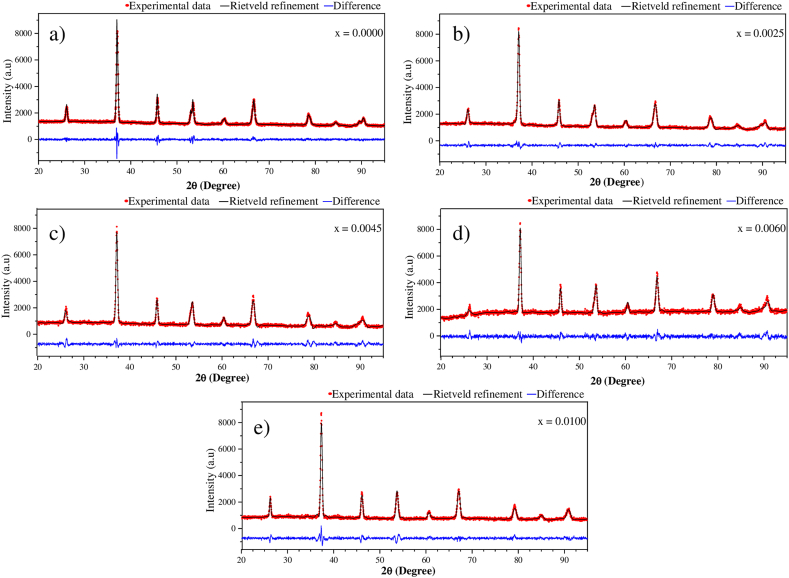
Rietveld refinement of XRD patterns of the powder samples sintered at 1200 °C during 6 h in air atmosphere with different concentrations of x.

The structural evolution of the Ba_1-3x_La_2x_Ti_1-3x_Bi_4x_O_3_ solid solutions was studied through changes in the volume of the unit cell and its lattice parameters (*a* and *c*). The Rietveld refinement method was used to calculate these structural parameters, and the refined profiles are shown in Fig. 3. The x = 0.05 composition was excluded from the refinement results because it contained a secondary phase in its structure, unlike the other compositions which consist exclusively of BaTiO_3_. The computed lattice parameters and the reliability factors that characterize the fitting quality, i.e., the goodness of fit (χ^2^) and profile Rfactor (Rp) are shown in Table 1. The Rietveld refinement results confirmed the formation of pure tetragonal structure for 0.0 ≤ x ≤ 0.006 and pure cubic structure for x = 0.01. It was observed that the structural parameters for the tetragonal ferroelectric BaTiO_3_ samples in which the content of La^3+^ and Bi^3+^ (mol%) was 0.0025 ≤ x ≤ 0.006 experienced a slight contraction compared to the structural parameters for the undoped BaTiO_3_ sample (x = 0.0). This decrease in the lattice parameters was attributed to the partial substitution of La^3+^ and Bi^3+^ ions within the BaTiO_3_ structure. The ionic radius of the dopants La^3+^ and Bi^3+^ are 1.06 Å and 1.20 Å respectively, which are much larger than the ionic radius of Ti^4+^ ions (0.61 Å) but are comparable with that of Ba^2+^ ions (about r = 1.42 Å). Fig. 3 presents the intensities in arbitrary units for each of the XRD spectra, revealing that the intensities are comparable across the samples. This outcome was anticipated, given that all samples were collected over a uniform period of 60 min.

**Table 1:** Rietveld refinement results for the Ba_1-3x_La_2x_Ti_1-3x_Bi_4x_O_3_ samples sintered at 1200 °C during 6 h in air atmosphere.

x	Lattice parameters		Tetragonality c/a	Parameters characterizing the refinement quality		Unit cell volume (Å^3^)	Theoretical Density (g/cm^3^)	Crystalline structure
	a = b (Å)	c (Å)		χ^2^	Rp (%)			
**0.0**	3.9947	4.0279	1.0083	1.80	2.88	64.28	6.296	Tetragonal
**0.0025**	3.9820	4.0166	1.0087	1.66	2.84	63.69	6.114	Tetragonal
**0.0045**	3.9811	4.0101	1.0073	1.86	3.62	63.56	5.914	Tetragonal
**0.006**	3.9772	4.0050	1.0070	1.56	3.89	63.35	5.824	Tetragonal
**0.01**	3.9811	3.9811	1	1.80	3.36	63.10	5.652	Cubic

Consequently, in the final ceramics in an equilibrium state La^3+^ and Bi^3+^ ions should preferentially substitute in the Ba^2+^ sites, and due to the difference in valence; the substitution is compensated by either, free electrons or cation vacancies [23,38,39]. A decrease in the lattice parameters is expected, as the ionic radius of the dopants is smaller than that of Ba^2^⁺. Table 1 shows the unit cell volume evolution (*V)* for each synthesized sample. In this case, was observed that all the compositions presented a contraction in the unit cell volume compared to the undoped BaTiO_3_ sample, x = 0.0, obviously because of the decrease in the lattice parameters as mentioned above. This observation is consistent with literature results for BaTiO_3_ doped with La³⁺ and Bi³⁺ individually, where it has been observed that the 2θ angle of the diffraction peak increased gradually, indicating a decrease in the volume of the unit cell [40,41]. The theoretical densities of the doped BaTiO_3_ samples are shown as a function of La^3+^ and Bi^3+^ content in Table 1. The presence of La^3+^ and Bi^3+^ ions reduce the theoretical density of the doped ceramics. The density reaches its lowest value as the dopant content is close to its solubility limit in BaTiO_3_.

In the fourth column of Table 1, the evolution of the tetragonality ratio (*c/a*) of the system is presented, which is associated with the ferroelectric properties of the ceramic. A tetragonality ratio of 1 indicates a cubic phase, meaning the system no longer exhibits the ferroelectric properties characteristic of the tetragonal structure [39]. An increase in the tetragonality ratio is observed for the sample with x = 0.0025 compared to the undoped sample, rising from 1.0083 to 1.0087, thus surpassing the minimum tetragonality value (1.008) required by the MLCs industry. However, the tetragonality ratio was decreased at concentrations x = 0.0045 and x = 0.006. A tetragonality ratio of 1 was found for the sample with x = 0.01 of La^3+^ and Bi^3+^ (mol%), indicating a cubic phase. This finding is consistent with the XRD results shown in Fig. 2, where the beginning of a transition to the cubic phase was observed after the sample x = 0.006 of La^3+^ and Bi^3+^ (mol%). This is evident as one of the characteristic peaks of BaTiO_3_, located at position 2θ ≈ 53.49 in the tetragonal phase, begins to decrease.

### 3.2 Morphology and microstructure

**Fig. 4:**
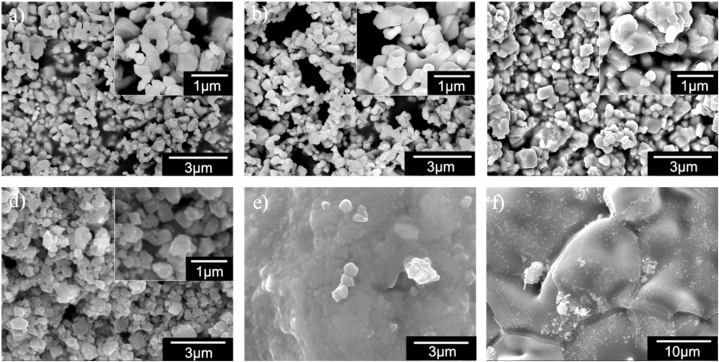
HRSEM micrographs of Ba_1-3x_La_2x_Ti_1-3x_Bi_4x_O_3_ ceramics for (a) x = 0.0, (b) x = 0.0025, (c) x = 0.0045, (d) x = 0.006, (e) x = 0.01, (f) x = 0.05.

The HRSEM micrographs of doped BaTiO_3_ ceramics sintered at 1200 °C for 6 h are shown in Fig. 4. The HRSEM micrographs for samples with x = 0.0, 0.0025, 0.0045, and 0.006 (mol %) were obtained at 10,000x and 20,000x magnification. The micrographs for the samples with x = 0.01 and x = 0.05 were acquired at 10,000x and 5,000x magnification, respectively. The microstructure of BaTiO_3_-based powders for the samples x = 0.0, 0.0025, 0.0045, and 0.006 La^3+^ and Bi^3+^ (mol %) consists of particle agglomerates of varying sizes and morphology and it can be observed that the dopants (La^3+^ and Bi^3+^) did not drastically modify the microstructure at these concentrations. The particles generally presented a faceted shape, which is distinctly for powders of doped and undoped BaTiO_3_ [9]. On the other hand, in samples with dopant concentrations higher than 0.01 (mol%), the development of a second phase took place which makes it difficult to observe the grains of BaTiO_3._ The above was attributed to the fact that Bi_2_O_3_ is a low melting point additive, whose melting point is 820 °C. It forms a liquid phase and promotes solid-state reaction during the sintering process [42].

**Fig. 5:**
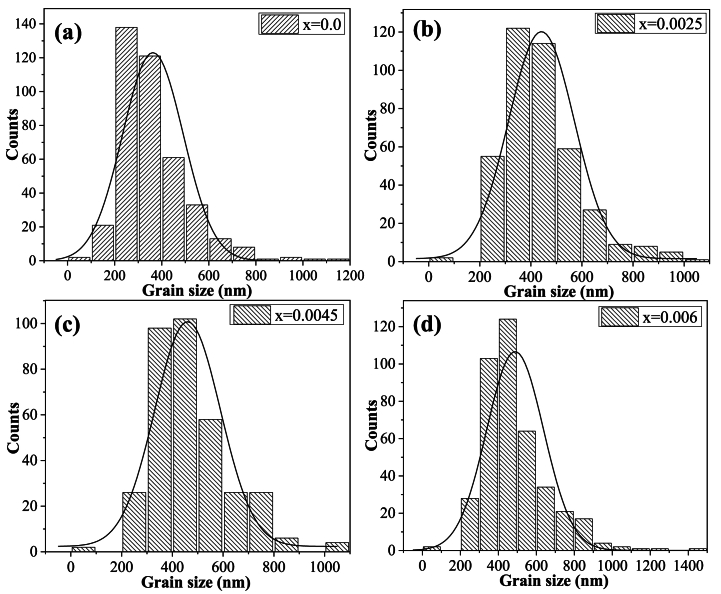
Histograms for grain size of Ba_1-3x_La_2x_Ti_1-3x_Bi_4x_O_3_ ceramics for (a) x = 0.0, (b) x = 0.0025, (c) x = 0.0045, (d) x = 0.006.

Microscopy is a technique that allows us to observe and measure individual particles. In this study, ceramography, thought of as the metallography of ceramics was used to measure the size and shape of grains. The size and microstructure of grains depend on the processing methods used to create the ceramic. The mean grain diameter was determined using the Feret measurement method with ImageJ software, which allows for precise individual grain measurements [22]. The Feret diameter is defined as the distance between two parallel tangents drawn along the edges of a grain in a specific direction. This diameter can be measured in multiple directions and is typically reported as the average diameter across these orientations. In this study, the Feret diameter was calculated in four different directions for each grain. A total of 400 grains were measured in HRSEM images to determine the mean grain diameter for compositions in the range of 0.0 ≤ x ≤ 0.006. Table 2 presents the mean diameter and standard deviation for each sample, while Fig. 5 illustrates the results as histograms with fitted normal curves for each case.

**Table 2:** Mean grain diameter and standard deviation of Ba_1-3x_La_2x_Ti_1-3x_Bi_4x_O_3_ solid solutions with different concentrations of x.

x	Mean (nm)	Standard Deviation (nm)	Aspect Ratio (A_R_)
**0.0**	362.5	143.8	0.7725
**0.0025**	441.4	145.2	0.7653
**0.0045**	475.5	151.0	0.7899
**0.006**	488.3	171.2	0.7836

From the mean size of the grains in each sample, it could be concluded that the grain growth was increased as the Bi^3+^ and La^3+^ content was increased. Other authors have reported that the particle size of La-doped BaTiO_3_ decreased with increasing dopant concentration [43]. However, it has also been reported that the grain size of Bi-doped BaTiO_3_ was increased with increasing dopant at low concentrations, as the samples studied in this work [12]. Then we can conclude that the grain growth in the samples with x = 0.0025, 0.0045, and 0.006 was mainly due to the addition of Bi^3+^ as a dopant. The increase in the standard deviation can be observed in Table 2 for the samples x = 0.0025, 0.0045, and 0.006, where a bimodal grain growth is presented with small grains and large grains coexisting in the sample.

The shape of grains can be quantified statistically using dimensionless ratios called shape factors. These ratios include axial lengths, areas, perimeters, and moments of grain shapes. 'Aspect ratio' refers to the ratio of the largest diameter of a grain to its smallest diameter, which can be calculated as follows:

(3) $$A_{R}=\frac{d_{L}}{d_{s}}$$

where *A*_*R*_ is the aspect ratio, *d*_*L*_ is the largest diameter and *d*_*S*_ is the smallest diameter. This ratio is presented in Table 2 and shows that the aspect ratio of the doped ceramics decreased with increasing doping concentration Bi^3+^ and La^3+^ (x = 0.0025), and subsequently increased from respect to undoped BaTiO_3_, being maximum at x = 0.0045.

Conventional quantitative microanalysis is a technique used to determine the elemental composition of small volumes of materials, typically at the micrometer scale. In this technique, HRSEM is employed to utilize the X-rays emitted from a specimen bombarded by a finely focused electron beam to identify the elements present in the material. X-rays can be generated from conventional flat-polished samples, depending on the initial electron beam energy and the atomic number of the elements, from volumes with linear dimensions as small as 1 μm. This enables the analysis of volumes as small as 10^−12^ cm^3^. The detection limit for a specific element in terms of mass is approximately 10^−14^ to 10^−15^ g in the HRSEM [44]. In this study, energy-dispersive X-ray spectroscopy (EDS) is utilized as a microanalysis technique to assess the presence of dopant elements in the samples and to observe their evolution as the amount of dopant increases.

**Fig. 6:**
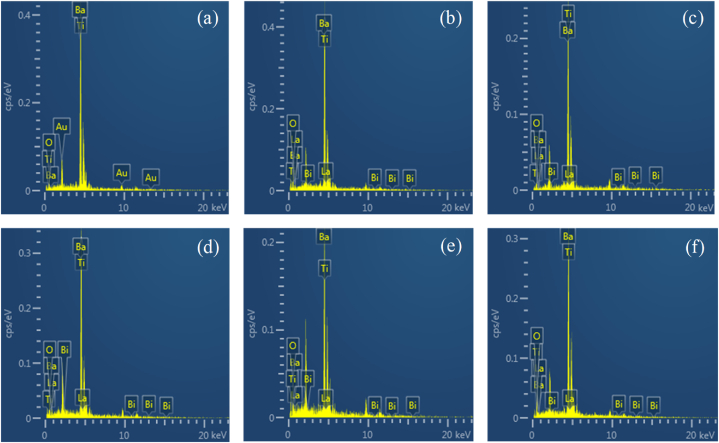
Punctual microanalysis of Ba_1-3x_La_2x_Ti_1-3x_Bi_4x_O_3_ ceramics for (a) x = 0.0, (b) x = 0.0025, (c) x = 0.0045, (d) x = 0.006, (e) x = 0.01, (f) x = 0.05.

Fig. 6 shows the EDS spectra of the sintered samples. These spectra clearly reveal the presence of Ba, Ti, and O, while La and Bi are barely detectable due to their low concentrations in the samples. Fig. 6(a) indicates the presence of Au, which is used as part of the sample preparation technique to prevent charge buildup on the sample surface that could distort the HRSEM image. In this case, Au was employed for coating using a sputter coater. However, the presence of gold was not considered in the semi-quantitative atomic percentage calculations of the elements.

Table 3 summarizes the semi-quantitative compositions obtained by HRSEM-EDS for all samples. The EDS analysis revealed that the doped BaTiO_3_ ceramics (0.0 ≤ x ≤ 0.05) contained Ba, Ti, O, La, and Bi elements near their surfaces. No other elements were detected. It was observed that the concentrations of La and Bi increased with increasing x, which is consistent with expectations.

**Table 3:** Semi-quantitative chemical compositions (HRSEM-EDS) of powders sintered at 1200 °C, 0 ≤ x ≤ 0.05 (mol%).

x	Atomic %				
	Ba	Ti	O	Bi	La
**0.0**	53.78	23.24	22.98	0	0
**0.0025**	47.52	23.07	28.56	0.23	0.62
**0.0045**	49.46	24.33	24.8	0.35	1.06
**0.006**	50.42	21.61	25.68	0.54	1.75
**0.01**	49.14	21.35	25.29	1.2	3.02
**0.05**	51.45	17.86	20.46	3.51	6.72

**Fig. 7:**
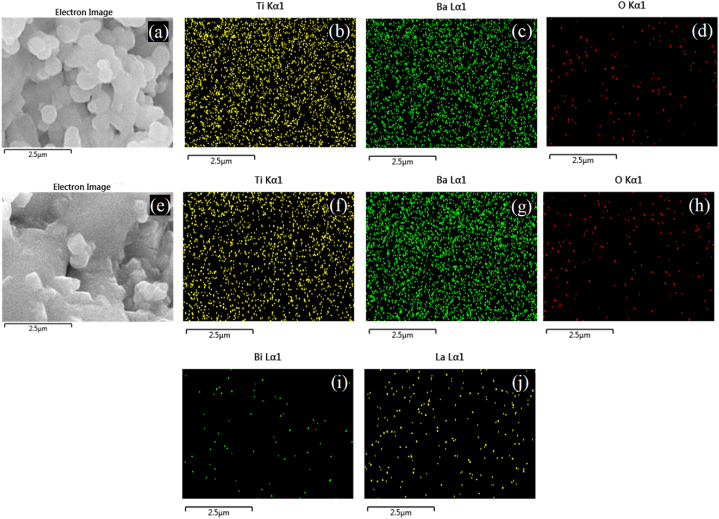
Elemental distribution mapping analysis of Ba_1-3x_La_2x_Ti_1-3x_Bi_4x_O_3_ solid solutions with x = 0.0, (a) Electron image, (b) Ti, (c) Ba, and, (d) O and x = 0.0045, (d) Electron image, (e) Ti, (f) Ba, (g) O, (h) Bi, and (i) La.

Additionally, Fig. 7 presents the results of elemental distribution mapping for x = 0.0 (a, b, c, d) and x = 0.0045 (e, f, g, h, i, j). These images further confirm the homogeneous incorporation of dopant ions into the BaTiO_3_.

## 4 Conclusions

In this work, the synthesis and structural evolution of La^3+^ and Bi^3+^ doped BaTiO_3_ solid solutions were analyzed, focusing on the study of phase transformations in the unit cells and the formation of secondary phases. The XRD patterns obtained for 0.0 ≤ x ≤ 0.006 corresponded to the diffraction pattern of BaTiO_3_ in the tetragonal phase at room temperature (JCPDS 961525438). A phase transformation from tetragonal to cubic symmetry was observed at x = 0.0100 (JCPDS 965910150). At x = 0.0500, a secondary phase, identified as BaBiO₃ was detected, which has been explored for its potential applications in positive temperature coefficient of resistivity (PTCR) devices. These results demonstrated that La³⁺ and Bi³⁺ ions can be incorporated into the BaTiO₃ lattice, with a solubility limit of x = 0.05 for the samples studied. It was determined that the concentration of La^3+^ and Bi^3+^ in solid solutions of the type Ba_1-3x_La_2x_Ti_1-3x_Bi_4x_O_3_ has significant effects on the *a* and *c* lattice parameters, which decrease compared to pure BaTiO_3_ in the tetragonal phase. The sample with x = 0.0025 was found to exhibit the maximum tetragonality among the samples studied, with a value of 1.0087, surpassing the minimum tetragonality value (1.008) required by the multilayer capacitor (MLC) industry. This finding fulfills one of the primary objectives of this work: to enhance the tetragonality of BaTiO₃ through doping. High-resolution scanning electron microscopy (HRSEM) analysis revealed that the microstructure of BaTiO₃-based powders consists of particles and agglomerates of varying sizes and faceted shapes. The observed particle distributions indicated that grain growth increases with higher dopant content, and an increase in the standard deviation of grain size with increasing dopant concentration suggests bimodal grain growth.

## CRediT authorship contribution statement

**M.I. Valenzuela-Carrillo:** Writing – original draft, Investigation, Formal analysis. **M. Pérez-Labra:** Writing – review & editing, Methodology, Formal analysis, Conceptualization. **F.R. Barrientos-Hernández:** Resources, Methodology, Investigation. **J.A. Romero-Serrano:** Resources, Methodology. **M. Reyes-Pérez:** Resources, Methodology. **A. Cruz-Ramírez:** Resources, Methodology. **M.U. Flores-Guerrero:** Methodology, Investigation. **J.C. Juárez-Tapia:** Resources, Methodology.

## Declaration of competing interest

The authors declare that they have no known competing financial interests or personal relationships that could have appeared to influence the work reported in this paper.

## References

[1] Vijatović M.M., Bobić J.D., Stojanović B.D. (2008). History and challenges of barium titanate: Part II. Sci. Sinter..

[2] Atta N.F. (2016).

[3] Yasmin S. (2011). Effect of cerium doping on microstructure and dielectric properties of BaTiO_3_ ceramics. J. Mater. Sci. Technol..

[4] Hu D. (2020). Optimization the energy density and efficiency of BaTiO_3_-based ceramics for capacitor applications. Chem. Engin. J..

[5] Vijatović M.M., Bobić J.D., Stojanović B.D. (2008). History and challenges of barium titanate: Part I. Sci. Sinter..

[6] Rödig T., Schönecker A., Gerlach G.A. (2010). A survey on piezoelectric ceramics for generator applications. J. Am. Ceram. Soc..

[7] Cochran W. (1960). Crystal stability and the theory of ferroelectricity. Adv. Phys..

[8] Tsurumi T., Sekine T., Kakemoto H., Hoshina T., Nam S.-M., Yasuno H., Wada S. (2006). Evaluation and statistical analysis of dielectric permittivity of BaTiO_3_ powders. J. Am. Ceram. Soc..

[9] Buscaglia M., Buscaglia V., Viviani M., Nanni P., Hanuskova M. (2000). Influence of foreign ions on the crystal structure of BaTiO_3_. J. Eur. Ceram. Soc..

[10] Brajesh K., Kalyani A.K., Ranjan R. (2015). Ferroelectric instabilities and enhanced piezoelectric response in Ce modified BaTiO_3_ lead-free ceramics. Appl. Phys. Lett..

[11] Vijatovic M., Stojanovic B., Bobic J., Ramoska T., Bowen P. (2010). Properties of lanthanum doped BaTiO_3_ produced from nanopowders. Ceram. Int..

[12] Wu S., Wei X., Wang X., Yang H., Gao S. (2010). Effect of Bi_2_O_3_ additive on the microstructure and dielectric properties of BaTiO_3_-based ceramics sintered at a lower temperature. J Mater Sci Techno.

[13] Ali A.I., Kaytbay S.H. (2011). Electrical transport properties of La-BaTiO_3_. Mater. Sci. Appl..

[14] Zdorovetsa M.V., Kozlovskiya A.L. (2019). Study of the effect of La^3+^ doping on the properties of ceramics based on BaTiO_3_. Vacuum.

[15] Sareecha N. (2018). Electrical investigations of Bi-doped BaTiO_3_ ceramics as a function of temperature. Phys. B Condens. Matter.

[16] Dunbar T.D., Warren W.L., Tuttle B.A., Randall C.A., Tsur Y. (2004). Electron paramagnetic resonance investigations of lanthanide-doped barium titanate: dopant site occupancy. J Phys Chem.

[17] Hennings D.F. (2001). Dielectric materials for sintering in reducing atmospheres. J. Eur. Ceram. Soc..

[18] Yoon D. (2006). Tetragonality of barium titanate powder for a ceramic capacitor application. J. Ceram. Process. Res..

[19] Stojanovic B.D., Jovalekic C., Vukotic V., Simoes A.Z., Varela J.A. (2005). Ferroelectric properties of mechanically synthesized nanosized barium titanate. Ferroelectrics.

[20] Stojanovic B., Simoes A., Paiva-Santos C., Jovalekic C., Mitic V., Varela J. (2005). Mechanochemical synthesis of barium titanate. J. Eur. Ceram. Soc..

[21] Magdalinovic N., Trumic M., Trumuc M., Andric L. (2012).

[22] Merkus H.G. (2009).

[23] Kocserha I. (2023). Role of A-site (Sr), B-site (Y), and A, B sites (Sr, Y) substitution in lead-free BaTiO_3_ ceramic compounds: structural, optical, microstructure, mechanical, and thermal conductivity properties. Ceram. Int..

[24] Cumbrera F.L., Moshtaghioun B.M., Gómez-García D., Ortiz A.L. (2023). Quantifying structural disorder in spinels by X-ray diffractometry through constrained– restrained Rietveld refinements. Ceram. Int..

[25] Dhahri A., Belkahla A., Laifi J., Gouadria S., Elhadi M., Dhahri J., Dhahri E. (2022). Crystal structure and dielectric properties of the Ca/Y co-substituted BaTiO_3_. Inorg. Chem. Commun..

[26] Morrison F.D., Sinclair D.C., West A.R. (1999). Electrical and structural characteristics of lanthanum-doped barium titanate ceramics. J. Appl. Phys..

[27] Iriani c Y., Suherman B., Sandi D.K., Nurosyid F., Khairuddin, Handoko E., Faquelle D. (2024). Structural modification and dielectric property of Bi-doped BaTiO_3_ (Ba_1-x_Bi_x_TiO_3_) ceramics with co-precipitation technique. Integrated Ferroelectrics Int. J..

[28] Sleight A.W. (2015). Bismuthates: BaBiO_3_ and related superconducting phases. Physica C (Amsterdam, Neth.).

[29] Bouwmeester R.L., Brinkman A. (2021). BaBiO_3_ from single crystals towards oxide topological insulators. Reviews in Physics.

[30] Pei S., Jorgensen J.D., Dabrowski B., Hinks D.G., Richards D.R., Mitchell A.W., Jacobson A.J. (1990). Structural phase diagram of theBa_1−x_K_x_BiO_3_system. Phys. Rev. B.

[31] Bazhirov T., Coh S., Louie S.G., Cohen M.L. (2013). Importance of oxygen octahedra tilts for the electron-phonon coupling in K-doped BaBiO_3_. Phys. Rev. B.

[32] Tajima S., Uchida S., Masaki A., Takagi H., Kitazawa K., Tanaka S., Katsui A. (1985). Optical study of the metal-semiconductor transition inBaPb_1−x_Bi_x_O_3_. Phys. Rev. B.

[33] Mattheiss L.F., Hamann D.R. (1982). Electronic- and crystal-structure effects on superconductivity in theBaPb_1−x_Bi_x_O_3_system. Phys. Rev. B.

[34] Luo Y., Liu X., Li X., Liu G. (2006). PTCR effect in BaBiO_3_-doped BaTiO_3_ ceramics. Solid State Ionics.

[35] Yuan C.-L., Liu X.-Y., Zhou C.-R., Xu J.-W., Yang Y. (2011). Characterization of the BaBiO_3_-doped BaTiO_3_ positive temperature coefficient of a resistivity ceramic using impedance spectroscopy with Tc= 155 °C. Chin. Phys. B.

[36] Yao Z., Liu H., Liu Y., Wu Z., Shen Z., Liu Y., Cao M. (2008). Structure and dielectric behavior of Nd-doped BaTiO_3_ perovskites. Mater. Chem. Phys..

[37] Hernández Lara J.P., Pérez Labra M., Barrientos Hernández F.R., Romero Serrano J.A., Ávila Dávila E.O., Thangarasu P., Hernández Ramirez A. (2017). Structural evolution and electrical properties of BaTiO_3_ doped with Gd^3+^. Mater. Res..

[38] Dunbar T.D., Warren W.L., Tuttle B.A., Randall C.A., Tsur Y. (2004). Electro paramagnetic resonance investigations of lanthanide-doped barium titanate: dopant site occupancy. J. Phys. Chem. B.

[39] Martínez-López R., Perez-Labra M., Romero-Serrano J.A., Barrientos-Hernández F.R., Reyes-Pérez M., Valenzuela-Carrillo M.I., Dávila-Pulido G.I. (2023). BaTiO_3_ solid solutions co-doped with Gd^3+^ and Eu^3+^: synthesis, structural evolution and dielectric properties. J. Rare Earths.

[40] Tihtih M., Ibrahim J.F.M., Kurovics E., Abdelfattah M. (2020). Study on the effect of Bi dopant on the structural and optical properties of BaTiO_3_ nanoceramics synthesized via sol-gel method. J. Phys. Conf..

[41] Ianculescu A., Mocanu Z.V., Curecheriu L.P., Mitoseriu L., Padurariu L., Truşcă R. (2011). Dielectric and tunability properties of La-doped BaTiO_3_ ceramics. J. Alloys Compd..

[42] Yu Z., Sun K., Li L., Liu Y., Lan Z., Zhang H. (2008). Influences of Bi_2_O_3_ on microstructure and magnetic properties of MnZn ferrite. J. Magn. Magn Mater..

[43] Xinle Z., Zhimei M., Zuojiang X., Guang C. (2006). Preparation and characterization on nano- sized barium titanate powder doped with lanthanum by sol-gel process. J. Rare Earths.

[44] Goldstein J.I., Newbury D.E., Michael J.R., Ritchie N.W., Scott J.H.J., Joy D.C. (2017).

